# Accurate personalized survival prediction for amyotrophic lateral sclerosis patients

**DOI:** 10.1038/s41598-023-47935-7

**Published:** 2023-11-24

**Authors:** Li-Hao Kuan, Pedram Parnianpour, Rafsanjany Kushol, Neeraj Kumar, Tanushka Anand, Sanjay Kalra, Russell Greiner

**Affiliations:** 1 Department of Computing Science, University of Alberta, Edmonton, Canada; 2 Neuroscience and Mental Health Institute, University of Alberta, Edmonton, Canada; 3 Alberta Machine Intelligence Institute, Edmonton, Alberta Canada; 4 Division of Neurology, Department of Medicine, University of Alberta, Edmonton, Canada

**Keywords:** Amyotrophic lateral sclerosis, Computer science

## Abstract

Amyotrophic Lateral Sclerosis (ALS) is a rapidly progressive neurodegenerative disease. Accurately predicting the survival time for ALS patients can help patients and clinicians to plan for future treatment and care. We describe the application of a machine-learned tool that incorporates clinical features and cortical thickness from brain magnetic resonance (MR) images to estimate the time until a composite respiratory failure event for ALS patients, and presents the prediction as individual survival distributions (ISDs). These ISDs provide the probability of survival (none of the respiratory failures) at multiple future time points, for each individual patient. Our learner considers several survival prediction models, and selects the best model to provide predictions. We evaluate our learned model using the mean absolute error margin (MAE-margin), a modified version of mean absolute error that handles data with censored outcomes. We show that our tool can provide helpful information for patients and clinicians in planning future treatment.

## Introduction

### Amyotrophic lateral sclerosis

Amyotrophic lateral sclerosis (ALS) is a progressive neurodegenerative disease of adulthood with a mean age of onset in the late fifties to early sixties. The motor neurons in the brain, brainstem, and spinal cord are prime targets of the disease. However, non-motor regions can also be involved, including the frontotemporal lobes. As the illness progresses, weakness usually results in loss of function of the limbs, difficulty walking and speaking, and eventually breathing. The median survival following the onset of symptoms is 2–5 years, and respiratory failure is commonly the reason for death. However, the interval between the onset of symptoms and death can range from a few months to more than ten years^1^. This range makes it impossible for clinicians to effectively counsel patients on advance care planning, or to select patients with specific survival characteristics for drug trials. While clinicians often employ some pharmacologic treatments (e.g., riluzole, edaravone, and AMX0035)^2^ in the early stages of the disease to slow disease progression and extend survival time, the effects are modest and no treatment is known to stop the advancement of the disease. Although no one has found a way to use routine clinical brain MRI (magnetic resonance imaging) scans for diagnostic or prognostic purposes, there is a promise that advanced MRI of the brain and spinal cord might be helpful. Notably, cortical thinning of motor and extra-motor regions as a consequence of the pathological changes related to cerebral degeneration is measurable using T1-weighted structural MR images.^3^

### Survival prediction for ALS

This paper provides a way to predict the time until composite respiratory failure, as this will help patients make decisions about future treatment or care—e.g., move to hospice if death in under 3 months, or decide about treatment based on a quantitative estimate of the survival times for different options. Moreover, many patients also want an estimate of their specific survival time. Below we list several earlier survival analysis approaches to this task, and note that, while each provide some useful information, none predict the actual time until death/failure.

Querin et al.^4^ employed a multivariate Cox regression model for predicting survival of ALS patients using clinical, spinal cord MRI and diffusion tensor imaging (DTI) data and reported significant association of fractional anisotropy and magnetization transfer ratio features. However, their study aims to identify important covariates but does not report prediction-related statistics. Lunetta et al.^5^ proposed an ALS-Survival Score (ALS-SS) system using multivariate Cox model over some clinical features and reported a combination of age, ALS Functional Rating Scale-Revised (ALSFRS-R) score, and body mass index factors are relevant to survival. The ALS-SS system produces a risk score for each patient (which can be used to predict which patient will die first) but does not provide the expected length of survival itself. Many studies formulate the survival prediction task as a binary classification for a single time point, such as surviving one year or two years. Schuster et al.^6^ logistic ridge regression model used clinical and/or MRI features to predict 18-month survival, with prediction accuracy of 66.67% from clinical features, 77.08% from MRI features and 79.17% from both. Pfohl et al.^7^ learned generalized linear and random forest models over 38 clinical features to classify survival at different time points, starting from 30 days to 5 years. Another study successfully applied the non-linear dimension reduction technique, Uniform Manifold Approximation and Projection (UMAP)^8^, for 1-year survival analysis in ALS and claimed to achieve 94% accuracy^9^. Introna et al.^10^ investigated whether the slope of the King’s College ALS clinical staging (KC) system^11^ at the initial visit could predict survival in a cohort of ALS patients; they found the KC progression rate ($\Delta$KC) demonstrated an accuracy of 92%, 85%, and 83% in predicting survival at one year, two years, and three years, respectively. Overall, the performance for single time point prediction is promising for some specific time points. However, those studies appear to ignore censored individuals (defined in the next section), which can be a large proportion. Also, the time points being predicted varies for different studies. Our individual survival curve model (described in the next section) provides survival probability for all future time points, and also explicitly deals with censored patients.

Some studies predict the survival time by describing the task as classifying event occurrence into multiple time windows. Van der Burgh et al.^12^ applied a deep neural network to eight clinical features, along with some imaging features from diffusion-weighted and T1-weighted MR images, to produce a model that could classify ALS patients as short, medium or long survivors. They found that just using those eight clinical features (or just the MRI characteristics) could not achieve robust performance, but the combined features obtained 84.4% classification accuracy. Corrado et al.^13^ proposed machine learning methods capable of addressing competing risks and censoring for the Intelligent Disease Progression Prediction (IDPP) challenge dataset.^14^ These ML techniques produce an average C-index of around 0.70 and 0.74, utilizing data from the first visit and 6 months later, respectively, to predict competing risks such as non-invasive ventilation (NIV), percutaneous endoscopic gastrostomy (PEG) and death. They report 0.86 specificity but low sensitivity for predicting the time of event occurrence. Although knowing the event time window can help us narrow down the estimated event time, the size of the time window might not meet the needs of clinicians (e.g., van der Burgh et al.^12^ use only three time windows). Also, if the data has censored instances, any model that does not handle censoring (e.g., the model used by van der Burgh et al.^12^) might introduce bias. In our approach, the learned model predicts the individual survival curves. Westeneng et al.^15^ use the multivariable Royston-Parmar survival model (Royston et al.^16^) to predict individual survival curves and report a C-index of 0.78. However, they did not report any event time prediction error measurement.

The papers above describe various approaches to survival prediction—e.g., produce a risk score, or a 1-year survival probability, etc. While useful for some tasks, we note that none actually estimates how long a person will survive. Below we provide a way to produce such personalized estimates. As our goal is a model that minimizes the difference between predicted time and true survival time, we therefore evaluate proposed models by their mean absolute error (MAE-Margin; see section “MAE-margin”) based on predicted median survival times. (Note that it is possible that a model could have a good C-index, but a bad MAE-Margin, and vice versa; similarly for 1-year survival probability vs MAE-Margin; etc.^17^)

**Figure 1:**
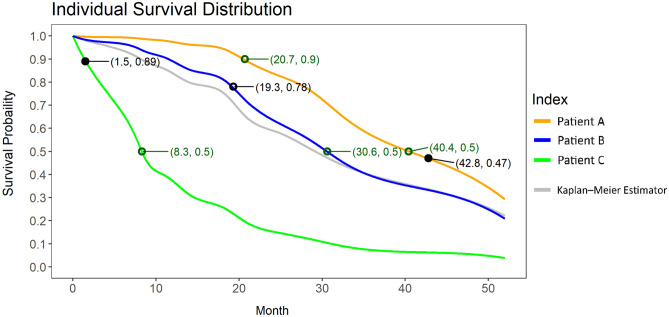
Real examples of individual survival curves for multiple patients predicted by our learned model. An individual survival curve provides the probability of survival until time *t*, for all $t \ge 0$. The empty green circles are some reference points on the curve, which can be used as predictions (e.g., the green circle on the blue line shows the prediction that Patient B has a 50% chance of surviving at least 30.6 months.) The x-coordinate of each solid black dot is the ground truth death time, and the empty black dot is the censoring time for that patient—e.g., patient A actually died at 42.8 months, and Patient B is censored at 19.3 months. The grey curve is the Kaplan–Meier estimator,^18^ which is a group statistic that includes all the patients.

### Personalized survival prediction

We view survival prediction as learning a model that can estimate the time until an event, for each individual. This “time to an event” task resembles regression - given a description of a patient, predict a non-negative real value (his/her time to death). Learning a survival model is more complicated, as the training data typically includes censored training instances, which provide only a lower bound on the survival time. (For example, imagine a patient left the study after 220 days and is then lost to follow-up. We know this patient lived for at least 220 days, but we do not know whether she lived 221 or 1000 days or 30 years.) As 45% of the patients in our data are censored, it is important that the process for learning our survival prediction models incorporates these censored training instances to avoid bias^19^; note our evaluation must also deal with such censored labels. The field of survival prediction (i.e., survival analysis) deals with this kind of censored data.

A single time-point binary classification (e.g., the probability that a patient will die before 100 days) might not be enough to tell the full story of a patient’s survival. A more recent approach provides an “individual survival distribution” (ISD)^20^ for each patient, which is a survival probability for all future times (i.e., a survival distribution) specific to this individual; see Fig. 1. The survival curve for a patient provides the probability that this patient will live until at least time *t* (i.e., survival probability) at each future time point $t \ge 0$. For example, the model shown in Fig. 1 predicts that patient A has a 90% probability of living at least $t = 20.7$ months and 50% probability of living at least $t = 40.4$ months, etc. We also see that this model makes different predictions for Patients A, B, and C.

Here, we consider the time until the composite respiratory failure event occurs: death, tracheostomy, or use of non-invasive ventilation for more than 23 hours per day. The composite respiratory failure, which is related to the use of surrogates to sustain survival, is widely used in ALS survival research.^10,15,21^ We develop a SuperLearner that learns a model that can predict ISDs for each ALS patient using the patient’s clinical information and cortical thickness extracted from the patient’s MR image. The SuperLearner selects the best survival prediction models from several candidates to provide predictions. Our SuperLearner aims to achieve the best MAE-margin (mean absolute error margin, defined in section “MAE-margin”), which is a variant of MAE (mean absolute error) that can deal with censored data.

## Results

### Machine learning SuperLearner for ALS survival prediction

We develop a machine learning SuperLearner that produces a survival model that can predict the time until the composite respiratory failure event, using the patient’s clinical and MR image features. Our SuperLearner considers several different hyperparameters that differ by the type of base ISD learner, the features used to describe each patient, and whether it applied a feature selection process to reduce the set of features. The choice of these hyperparameters is decided during the training process using internal cross-validation of the training data. Our overall learner first uses internal cross-validation to identify the best hyperparameter setting, then runs that best learner over the entire dataset to produce our final learned model. Note this learned model will provide predictions for novel instances. In addition to producing this final model, we also ran external five-fold stratified cross-validation to evaluate our final model.

### MAE-margin

We use mean absolute error margin (MAE-margin)^20^ to evaluate our learned model. This measurement is based on the mean absolute error (MAE), which measures the difference between predicted and actual event times. For example, if the model predicts the patient will die on day 7 and the true event time is day 10, the absolute error is 3 days. Of course, this assumes we know when the patient actually died. Note the simplified approach of just testing on the *uncensored* instances can lead to biased results because patients whose events happen early are less likely to be censored (e.g., a patient who died on day 1 is less likely to be censored than a patient who died on day 1000). The MAE-margin is a modification of MAE that handles censored data by estimating the “true event time” for each censored instance. We estimate the true event time as the mean of the Kaplan–Meier survival curve, conditioned on living (at least) until the censoring time. We use the median of each patient’s survival curves as that patient’s predicted event time. For detailed descriptions of MAE-margin, see Supplementary Information Sect. B.

Our learned model shows an MAE-margin of 14.2 (95% confidence interval ± 2.3) months. Note this MAE-margin is 22.3% of the longest time (either censored or uncensored) in our dataset, which is 78.8 months. Our MAE-margin is better than the baseline error of the Kaplan–Meier estimator, which is 17.4. The Kaplan–Meier estimator does not consider covariates, so the predictions are the same for all the patients. Although we are seeking the model that minimizes the MAE-margin, note its C-index (concordance index) is 0.70, showing that our SuperLearner can discriminate the risk of different patients (C-index measures the discrimination ability at all times. See detailed descriptions in Supplementary Information Sect. B).

### SuperLearner hyperparameters setting

Our SuperLearner considers several learners. During the training process, we first run internal cross-validation using the training data to select the best base learner and hyperparameters setting. The SuperLearner considers four base survival prediction algorithms: (1) accelerated failure time,^22^ (2) Cox proportional hazard^23^ with Kalbfleisch-Prentice estimator,^24^ (3) multitask logistic regression (MTLR),^25^ and (4) random survival forest^26^—all four models are described in Supplementary Information Sect. A. Our SuperLearner considered two feature selection options: (1) multivariate cox feature selection, (2) no feature selection. It also considers three sets of input features: (1) clinical features, (2) image features, and (3) both clinical and image features. Altogether, it considers $4 \times 2 \times 3 = 24$ different combinations. The SuperLearner first uses internal cross-validation (5 folds) to select the best combination, and then runs this combination over the entire training set to produce the final model. The results from the internal cross-validation choose the hyperparameter setting to be the MTLR algorithm, multivariate cox feature selection, and using only the clinical features. The L2 regularization constant for MTLR is also tuned and the optimal value is 1.

## Discussion

Our learned survival prediction model predicts an individual survival curve (ISD) for each ALS patient. The survival distribution can be more useful than single-time prediction (e.g., Surviving 1 year) when planning future patient treatment because the user can query the predicted survival time for arbitrary survival probabilities. In our analysis above, to compute MAE-Margin, we use the median of the survival curve as the predicted survival time (50% survival probability). Still, clinicians can query other probabilities, such as 90% survival probability, according to their needs (see Fig. 1). For example, imagine the time for supportive care needs to be reserved at time zero. According to the local healthcare policy, the clinician should plan the service at the 90% survival probability time because the policy targets a service utilization rate of 90%. The patient will be 90% chance alive at that time point to use the service. Our learned model gives a personalized prediction specific to this patient rather than only telling the patient the average survival time of all ALS patients. Our learned model performs better than the baseline Kaplan–Meier estimator, which implies that our learned model can effectively discriminate the differences between the patients and provide personalized predictions. The disease progression varies greatly for ALS patients, so it is important to consider their condition when planning future treatments. Our learned model considers the personal descriptions of the patient when giving survival curve predictions.

As a quick example of the MAE-margin approach, recall that Fig. 1 shows the survival curves of two patients, which we see are very different: Patient A’s median survival time is 40.4 months, while Patient C’s is 8.3 months. We see this is fairly accurate, as Patient A actually died at 42.8 months and Patient C at 1.5 months. This means the MAE (for these two patients) is $\frac{1}{2} (|40.4-42.8|+|8.3-1.5|) = 4.6$. By contrast, the median for the Kaplan–Meier estimator is 29.2, which means its error was $\frac{1}{2} ( |29.2-42.8|+|29.2-1.5|) = 20.65$. Patient B is censored, so the survival time is missing. The MAE-margin estimates the survival time by considering Patient B’s censored time 19.3 and the Kaplan–Meier estimator. Patient B is estimated to die at 36.5 months, and we treat this time as the true event time when calculating MAE-margin. Note that the Kaplan–Meier estimator does not use any covariate and is unrelated to the model being evaluated.

Several studies^10,15^ have used the definition of composite respiratory failure event, and unfortunately, they did not use MAE to measure their prediction model. One nice feature of ISDs is that we can use them for other tasks and evaluate them using their associated metric. Here, as Introna et al.^10^ predict one year, two years, and three years of survival, we also predict survival for these time points. For this binary classification task, censored patients are handled by our survival prediction model during the training process, and patients censored before the time point of interest are excluded during evaluation. Our learned model reports an accuracy of 0.77, 0.57, and 0.85 for one year, two years, and three years of survival. Westeneng et al.^15^ predict ISDs and report a C-index of 0.78. Our learned model has a C-index of 0.70. Although the above studies report better statistics than our model with their measurements, note that our model targets the MAE, representing a different aspect of the model’s performance.

The internal cross-validation in our tool chooses only to include clinical features. This result suggests that the cortical thickness in the MR image that we are providing cannot successfully improve the survival time predictions. However, a direct comparison of MAE-margin with external cross-validation between using the clinical features and using both feature-sets is not statistically significant in our experiment (two-tailed paired t-test p-value 0.61; see Supplementary Information Sect. C for detailed results). Further investigation is needed to verify these results.

## Methods

### Data description

#### Participant

Data was obtained from the Canadian ALS Neuroimaging Consortium (CALSNIC),^27^ a prospective multicentre longitudinal MRI-based observational study. Patients were selected with a symptom duration no more than 5 years and a diagnosis of one of {possible, probable, laboratory-supported probable, definite} ALS according to the Revised El Escorial Criteria^28^ The study cohort included 220 ALS patients (140 males and 80 females) with an average age of 59.7 years and an average symptom duration of 24.8 months. The CALSNIC study was conducted following the Declaration of Helsinki in addition to the approval of each participating site’s Health Research Ethics Board and informed written consent was obtained from the participants. The University of Alberta Health Research Ethics Board (HREB) approved the protocol presented in this study.

#### Clinical features

Our clinical features include the ALS Functional Rating Scale-Revised (ALSFRS-R) score, neurological examination, Edinburgh Cognitive and Behavioural ALS Screen (ECAS), finger and foot tapping, symptom duration, region of onset, years of education, handedness, age, and sex. The statistics for these training variables can be found in Supplementary Information Sect. D.

#### Image features

We extracted the cortical thickness from the MR image of the patients, then analyzed the cortical thickness using the FreeSurfer software (V.7.4.1, surfer.nmr.mgh.harvard.edu). Quality control was done before and after processing (segmentation and cortical thickness extraction) through the visual inspection of the T1-weighted MR images. We then extracted the mean cortical thickness of the 68 atlas-based ROIs for all the patients using automated pipeline.

### Data preprocessing

We use the following steps to transform the original data into a survival data format (including the survival times and censoring bits).

- Recruiting all the ALS patients diagnosed based on El Escorial criteria of possible, probable, or definite ALS from Canadian ALS Neuroimaging Consortium (CALSNIC) up until October 2021 (216 patients).
- Define each patient’s recruitment date as the date of his/her first clinical visit.
- For censored patients - i.e., patients with no respiratory or death event in our data - we used the difference between their recruitment date and the last hospital visit or follow-up as their censoring time. For patients with their death date or respiratory failure date recorded, we use those dates to compute their time to event (from recruitment). For ALS patients who opted for medical assistance in dying (MAID), we used their MAID dates as the last date for computing their censoring time, assuming that the time of MAID is independent of the patient’s survival . In total: 95 uncensored patients, and 121 censored patients, including 15 MAID patients.
- Remove all patients with censoring time of 0, which means the patient has no record after the first clinical visit (this removed 44 censored patients, leaving a total of 172 patients ).
- Describe each patient based on features from his/her first visit to the clinic.
- Remove features that were missing for more than 80% of the patients. (This removed 24 of the initial 138 clinical features. No feature was removed from MR images). The remaining missing variable values are replaced by mean imputation (7.9%).

**Figure 2:**
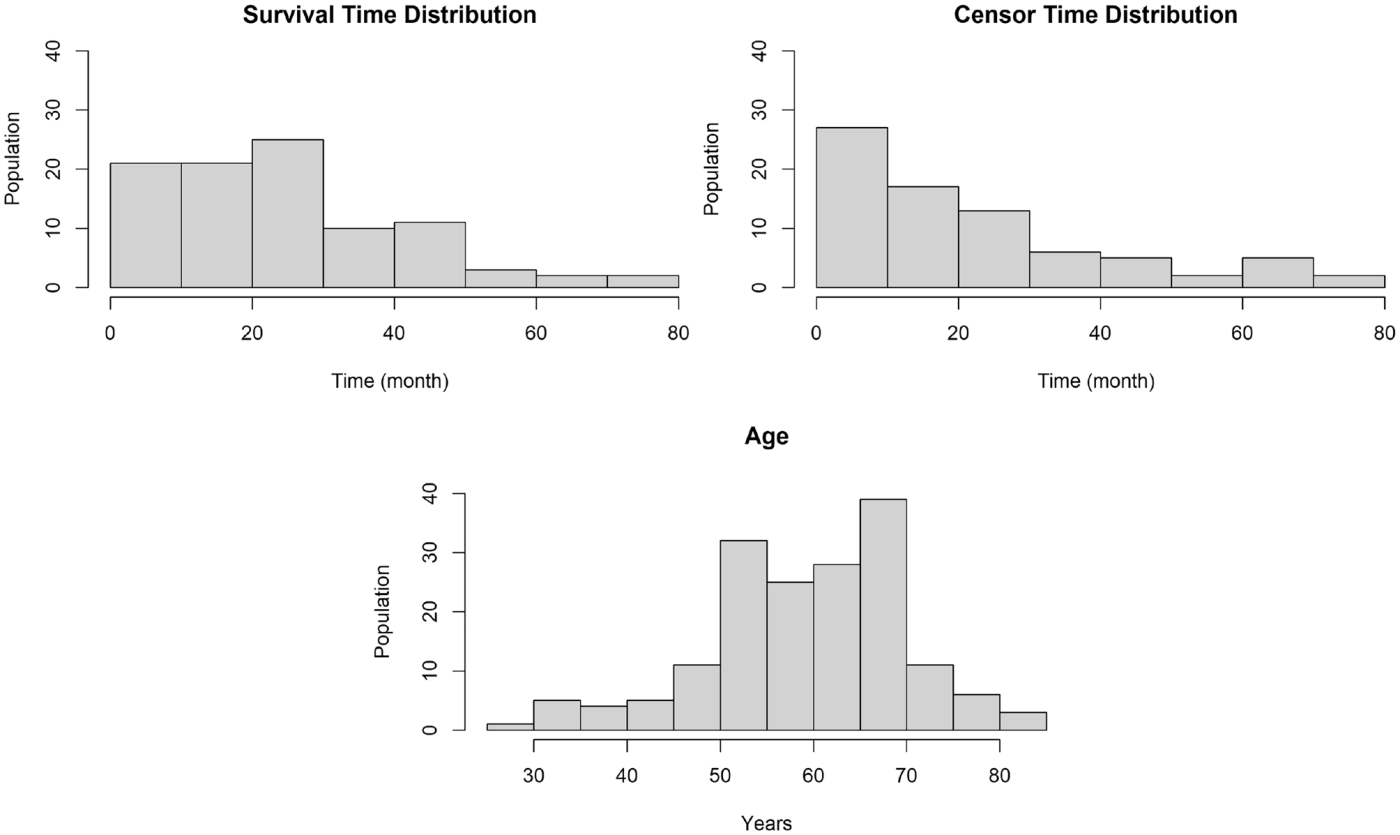
The survival time distribution for uncensored patients and censor time distribution for censored patients. The figure at the bottom is the age distribution of the patients.

After preprocessing, we have 172 ALS patients, each with 68 MR image features and 114 clinical features. The censoring rate for this dataset is 45%—i.e., 95 of the 172 patients experienced the composite respiratory failure event. Figure 2 shows the survival time, censor time, and age distribution of the data.

## Conclusion

We develop a machine-learning SuperLearner that can produce a learned model to predict the individual survival distribution for each ALS patient using that patient’s clinical features and cortical thickness from the MR images. Our target event is defined as the “composite respiratory failure” event. The SuperLearner considers multiple ISD models that differ by base algorithms, feature selection methods, and sets of input features. We evaluate the learned model of the SuperLearner by the MAE-margin to estimate the error of our survival time prediction. Finally, we show that the learned model provides personalized event time distribution prediction that can help patients and clinicians plan future treatments.

### Supplementary Information

Supplementary Information.

## Data Availability

The MRI and clinical data used in the analysis are available upon reasonable request to kalra@ualberta.ca, following the University of Alberta Ethics Committee’s data sharing and privacy rules.

## Code availability

Our codebase (not including data) is available on the GitHub repository https://github.com/kuan0911/ALS-survival-prediction-ISD.

## References

[1] Chio A (2009). Prognostic factors in als: a critical review. Amyotroph. Lateral Scler..

[2] Jaiswal MK (2019). Riluzole and edaravone: A tale of two amyotrophic lateral sclerosis drugs. Med. Res. Rev..

[3] Walhout R (2015). Cortical thickness in als: Towards a marker for upper motor neuron involvement. J. Neurol. Neurosurg. Psychiatry.

[4] Querin G (2017). Spinal cord multi-parametric magnetic resonance imaging for survival prediction in amyotrophic lateral sclerosis. Eur. J. Neurol..

[5] Lunetta C, Lizio A, Melazzini MG, Maestri E, Sansone VA (2016). Amyotrophic lateral sclerosis survival score (als-ss): A simple scoring system for early prediction of patient survival. Amyotroph. Lateral Scler. Frontotemp. Degen..

[6] Schuster C, Hardiman O, Bede P (2017). Survival prediction in amyotrophic lateral sclerosis based on mri measures and clinical characteristics. BMC Neurol..

[7] Pfohl SR, Kim RB, Coan GS, Mitchell CS (2018). Unraveling the complexity of amyotrophic lateral sclerosis survival prediction. Front. Neuroinform..

[8] McInnes, L., Healy, J. & Melville, J. Umap: Uniform manifold approximation and projection for dimension reduction. arXiv:1802.03426 (2018).

[9] Grollemund V (2020). Development and validation of a 1-year survival prognosis estimation model for amyotrophic lateral sclerosis using manifold learning algorithm umap. Sci. Rep..

[10] Introna A (2021). King’s college progression rate at first clinical evaluation: A new measure of disease progression in amyotrophic lateral sclerosis. J. Neurol. Sci..

[11] Roche JC (2012). A proposed staging system for amyotrophic lateral sclerosis. Brain.

[12] van der Burgh HK (2017). Deep learning predictions of survival based on mri in amyotrophic lateral sclerosis. NeuroImage: Clin..

[13] Corrado, P. *et al.* Multi-event survival prediction for amyotrophic lateral sclerosis. In *CEUR workshop proceedings*, vol. 3180, 1269–1276 (Faggioli G, Ferro N, Hanbury A, Potthast M, 2022).

[14] Guazzo, A. *et al.* Intelligent disease progression prediction: Overview of idpp@ clef 2022. In *International Conference of the Cross-Language Evaluation Forum for European Languages*, 395–422 (Springer, 2022).

[15] Westeneng H-J (2018). Prognosis for patients with amyotrophic lateral sclerosis: Development and validation of a personalised prediction model. Lancet Neurol..

[16] Royston P, Parmar MK (2002). Flexible parametric proportional-hazards and proportional-odds models for censored survival data, with application to prognostic modelling and estimation of treatment effects. Stat. Med..

[17] Qi, S. *et al.* An effective meaningful way to evaluate survival models. In Krause, A. *et al.* (eds.) *International Conference on Machine Learning, ICML 2023, 23-29 July 2023, Honolulu, Hawaii, USA*, vol. 202 of *Proceedings of Machine Learning Research*, 28244–28276 (PMLR, 2023). PMC12396822 40895293

[18] Kaplan EL, Meier P (1958). Nonparametric estimation from incomplete observations. J. Am. Stat. Assoc..

[19] Bouaziz, O. The effect of ignoring censoring in survival analysis: Theoretical and practical considerations. *University Paris Descartes and CNRS* (2010).

[20] Haider H, Hoehn B, Davis S, Greiner R (2020). Effective ways to build and evaluate individual survival distributions. J. Mach. Learn. Res..

[21] Miller, R., Mitchell, J. & Moore, D. Riluzole for amyotrophic lateral sclerosis (als)/motor neuron disease (mnd) in: Cochrane database of systematic reviews (2012). 10.1002/14651858.CD001447 11687111

[22] Wei L-J (1992). The accelerated failure time model: A useful alternative to the cox regression model in survival analysis. Stat. Med..

[23] Cox DR (1972). Regression models and life-tables. J. Roy. Stat. Soc.: Ser. B (Methodol.).

[24] Kalbfleisch JD, Prentice RL (1973). Marginal likelihoods based on cox’s regression and life model. Biometrika.

[25] Yu, C.-N., Greiner, R., Lin, H.-C. & Baracos, V. Learning patient-specific cancer survival distributions as a sequence of dependent regressors. *Adv. Neural Inf. Process. Syst.* **24** (2011).

[26] Ishwaran H, Kogalur UB, Blackstone EH, Lauer MS (2008). Random survival forests. Ann. Appl. Stat..

[27] Kalra, S. *et al.* The canadian als neuroimaging consortium (calsnic)-a multicentre platform for standardized imaging and clinical studies in als. *MedRxiv* (2020).

[28] Brooks BR (1994). El escorial world federation of neurology criteria for the diagnosis of amyotrophic lateral sclerosis. J. Neurol. Sci..

