# Supplementary material for: Accurate personalized survival prediction for amyotrophic lateral sclerosis patients
Source: Sci Rep. 2023 Nov 24;13:20713. doi: 10.1038/s41598-023-47935-7 (PMC10673879; doi:10.1038/s41598-023-47935-7)
Supplement: Supplementary file 1 — Supplementary Information. [file 41598_2023_47935_MOESM1_ESM.pdf]

# Supplementary Information

Supplementary Information for the manuscript "Accurate Personalized Survival Prediction for Amyotrophic Lateral Sclerosis Patients" by Li-Hao Kuan, Pedram Parnianpour, Rafsanjany Kushol, Neeraj Kumar, Tanushka Anand, Sanjay Kalra, and Russell Greiner.

## A Survival Prediction Algorithms

### A.1 Accelerated Failure Time

The accelerated failure time technique (AFT) [4] models the effect of features as the acceleration or deceleration of the event. The AFT model, first, assumes a survival distribution to fit the training data (we use the Weibull distribution in this paper). Then, the AFT model parameterizes the acceleration factor. The acceleration factor is a function of patients' covariates, and the acceleration factor controls the acceleration and deceleration of the patient's survival times (*e.g.*, old people die two times faster than young people). This AFT formulation can be expressed by:

$$S_i(t) \quad = \quad S_0(\, \exp(\theta \cdot x_i)\, t\, )$$

where $S_i(t)$ is the survival function for patient $i$, and $S_0(t)$ is the baseline survival function. $\exp(\theta \cdot x_i)$ is the acceleration factor, $x_i$ is the covariate for patient $i$, and $\theta$ is the trainable parameters for acceleration factor. Given a description of a new patient, the AFT model prediction the acceleration factor of that patient and produces an individual survival curve. In our implementation, we tuned the L2 regularization constant by internal cross-validation.

### A.2 Cox-KP

The Cox proportional hazard model (Cox-PH) [1] is one of the most popular algorithms to model survival data. The Cox-PH model estimates patient-specific risk scores that rank the relative risk of dying for patients (*e.g.*, patients with risk scores of 5 might die earlier than patients with risk scores of 4). In the Cox-PH model, the hazard ratio of any two patients is assumed to be constant over time. The hazard for a patient at time $t$ is the chance of failure at that time. If patient A's hazard is twice that of patient B in one month to three months, then the relationship is assumed to be the same for all the other times. This

relationship is called the proportional hazard assumption; it can be expressed as:

$$h_i(t) \quad = \quad h_0(t)\,\exp(\theta \cdot x_i)$$

where $h_i(t)$ is the hazard at time $t$ for patient $i$, and the $h_0(t)$ is the baseline hazard. The $\theta$ is the trainable parameters, and $x_i$ is the covariate vector for patient $i$. The hazard ratio is $exp(\theta \cdot x_i)$ which is not related to time, so the hazard ratio is proportional throughout all times. The vanilla Cox-PH model produces a risk score for each patient. The Cox-KP is the Cox proportional hazard model with the Kalbfleisch-Prentice estimator for baseline hazard. This extended Cox model produces a survival curve for an individual patient. In our implementation, we tuned the L2 regularization constant by internal cross-validation.

## A.3 Multi-task Logistic Regression

The multi-task logistic regression (MTLR) [5] is a discrete-time individual survival model that consists of a series of logistic regression models. The multi-task logistic regression first discretizes the future time into multiple disjoint intervals (*e.g.*, [0,100) days, [100,200) days). Then, the MTLR model builds a logistic regression model for each time interval to estimate the survival probability of that specific time interval. In order to formulate the dependency between different time intervals (the patient who already died can not come back to life), the MTLR model combines a series of logistic regression models from all the time intervals to compute the final prediction output. Given the description of a novel patient $x$, the resulting learned MTLR model predicts a survival probability distribution, $P(\,S > t \,|\, x\,)$. The survival probability distribution gives the probability that patient $x$ will live until at least time $t$ for each $t > 0$. In our implementation, we tuned the L2 regularization constant by internal cross-validation.

## A.4 Random Survival Forest

A random survival forest (RSF) [3] is built based on the well-known random forest regression algorithm. The random survival forest is modified to handle the censored survival data and is capable of computing individual survival distribution. This approach basically learns $M$ decision trees from the training data. Then, the training instances are partitioned into their leaf nodes. Once trained, a test instance is passed through all $M$ trees and reaches $M$ leaf nodes. The set of training instances associated with those leaf nodes will be collected and produce a single survival curve for each tree by using the Kaplan-Meier (KM) estimator. The $M$ survival curves from all trees are averaged together to produce the final predicted survival curve. In our implementation, we consider the implementation the same as Haider *et al.* [2] and tuned the number of trees and the nodesize using internal cross-validation.

# B Evaluation Metrics

## B.1 C-index

The C-index (concordance index) computes the proportion of correct risk ordering for all pairs of comparable instances. A pair of patients are correctly ordered if the patient with a higher predicted risk dies earlier than the other patients (*i.e.*, if the model claims patient A has a higher risk than patient B, then this model gets a "point" if, in fact, A dies before B.). A pair of patients is "comparable" if we can determine who died first. In the presence of censoring, a pair of patients is comparable if either they are both uncensored (event times are not missing) or one of the patients is censored later than the observed event time of the uncensored patient. The C-index is the proportion of correct ordering, so it is a real value between 0 to 1, where 1 means all comparable pairs are predicted correctly. C-index only measures the discriminative ability of a survival model and considers only a fraction of all pairs of patients (*i.e.*, only comparable pairs). For C-index, higher values indicate better models.

## B.2 MAE-Margin

The mean absolute error margin (MAE-margin) [2] is a modified version of the mean absolute error that includes censored instances. The mean absolute error evaluates the difference between predicted and true survival time, where we use the median of the patient's survival curves as the predicted survival time. Ignoring censored instances can lead to bias estimation because earlier event time is less likely to be censored. MAE-margin includes censored instances by estimating the true event time ($e_i$) for each censored instance as the mean of the Kaplan-Meier survival curve ($S_{\text{KM}}(\,t\,)$), conditioned on living until the censoring time. The estimated true event time $e_i$ can be written as:

$$e_{\text{margin}}(c_i) = \mathbb{E}_t[e_i \mid e_i > c_i] = c_i + \frac{\int_{c_i}^{\infty} S_{\text{KM}}(\,t\,)dt}{S_{\text{KM}}(\,c_i\,)}$$

where $c_i$ is the censoring time for patient $i$.

When calculating the MAE-margin, a re-weighting scheme is applied to each absolute error based on the censoring time to encode our confidence about the estimated event time. The weight is given as $\omega_i = 1 - S_{\text{KM}}(t_i)$ for censored instances. The longer the censoring time, the more confident we have about the estimated event time. The MAE-margin Equation, including both uncensored and censored data, can be written as:

$$\mathbb{E}_i[\text{MAE-margin}(\hat{t}_i, t_i, \delta_i)] = \frac{1}{\sum_{i=1}^{N} \omega_i} \sum_{i=1}^{N} \omega_i \left| [(1-\delta_i)\cdot e_{\text{margin}}(t_i) + \delta_i \cdot t_i] - \hat{t}_i \right|$$

where $\delta_i$ is the censored bit, and $t_i$ is the time for both censored and uncensored instances. $t_i$ is the true survival time if $\delta_i = 1$ and $t_i$ is the censoring time if $\delta_i = 0$. Lower MAE-margin values indicate better models.

| Input | MAE-margin | C-index |
|---|---|---|
| Image | 18.8 ± 1.2 | 0.52 ± 0.08 |
| Clinical | 14.4 ± 2.5 | 0.71 ± 0.07 |
| Image + Clinical | 14.2 ± 2.6 | 0.70 ± 0.09 |

Supplementary Table 1: Comparing MAE-margin for using different sets of input features. The second column shows the MAE-margin and standard deviation in months

# C Additional Results

## C.1 Image and Clinical Features

Supplementary Table 1 shows the MAE-margin for three learned models that use different sets of input features: (1) image features, (2) clinical features, and (3) both clinical and image features. The learner is the same superLearner described in this paper, except the set of input features is specified. Other hyperparameters are still selected by internal cross-validation. The evaluation is done by external five-fold stratified cross-validation.

# D Training Variables

Supplementary Table 2, 3, and 4 shows the mean and standard deviation of variables that we use to train our model. These variables will be normalized before training our model.

| Clinical Variables | Mean | Standard Deviation |
|---|---|---|
| Age | 59.97 | 10.65 |
| Sex | 66% male, 34% female | NA |
| Handedness | 83% right, 23% left, 4% ambidextrous | NA |
| YearsEd | 14.75 | 3.20 |
| Symptom Duration | 23.42 | 19.49 |
| Side_1st_MotorSymptomOnset | 53% right, 36% left, 14%bilateral | NA |
| MedicalExamination_Riluzole | 0.7 | 0.45 |
| ALSFRS TotalScore | 37.42 | 6.54 |
| Fingertapping Right avg | 43.36 | 17.65 |
| Fingertapping Left avg | 38.62 | 16.65 |
| Foottapping Right avg | 25.08 | 15.31 |
| Foottapping Left avg | 23.21 | 15.08 |
| UMN R | 2.375 | 1.53 |
| UMN L | 2.44 | 1.49 |
| UMN Burden w/o pseudo bubar score | 5.26 | 3.10 |
| LMN Right | 2.52 | 1.30 |
| LMN Left | 2.48 | 1.35 |
| LMN Burden | 5.88 | 2.85 |
| NE ElEscorial Diagnosis | 2.54 | 1.04 |
| ECAS ALSSpecific Total | 77.86 | 11.72 |
| ECAS TotalScore | 104.72 | 14.36 |
| ALSFRS slope | -0.70 | 0.74 |
| Region of onset: lower extremity | 0.45 | 0.49 |
| Region of onset: upper extremity | 0.36 | 0.48 |
| Region of onset: bulbar | 0.22 | 0.41 |
| Region of onset: bulbar speech | 0.17 | 0.37 |
| Region of onset: bulbar swallowing | 0.06 | 0.24 |

Supplementary Table 2: The statistics for some clinical variables. We did not show the entire list of the clinical variables that we use, but we listed the statistics for some variables that are more representative.

| Image Variables | Mean | Standard Deviation |
|---|---|---|
| LH_Thickness_bankssts | 2.38 | 0.22 |
| LH_Thickness_caudalanteriorcingulate | 2.41 | 0.22 |
| LH_Thickness_caudalmiddlefrontal | 2.46 | 0.19 |
| LH_Thickness_cuneus | 1.77 | 0.17 |
| LH_Thickness_entorhinal | 2.97 | 0.4 |
| LH_Thickness_frontalpole | 2.67 | 0.24 |
| LH_Thickness_fusiform | 2.55 | 0.21 |
| LH_Thickness_inferiorparietal | 2.35 | 0.18 |
| LH_Thickness_inferiortemporal | 2.67 | 0.19 |
| LH_Thickness_insula | 2.83 | 0.22 |
| LH_Thickness_isthmuscingulate | 2.23 | 0.19 |
| LH_Thickness_lateraloccipital | 2.05 | 0.16 |
| LH_Thickness_lateralorbitofrontal | 2.53 | 0.19 |
| LH_Thickness_lingual | 1.85 | 0.18 |
| LH_Thickness_medialorbitofrontal | 2.34 | 0.17 |
| LH_Thickness_middletemporal | 2.68 | 0.2 |
| LH_Thickness_paracentral | 2.27 | 0.21 |
| LH_Thickness_parahippocampal | 2.54 | 0.28 |
| LH_Thickness_parsopercularis | 2.48 | 0.19 |
| LH_Thickness_parsorbitalis | 2.62 | 0.21 |
| LH_Thickness_parstriangularis | 2.35 | 0.18 |
| LH_Thickness_pericalcarine | 1.52 | 0.18 |
| LH_Thickness_postcentral | 2.03 | 0.14 |
| LH_Thickness_posteriorcingulate | 2.32 | 0.19 |
| LH_Thickness_precentral | 2.4 | 0.22 |
| LH_Thickness_precuneus | 2.26 | 0.19 |
| LH_Thickness_rostralanteriorcingulate | 2.62 | 0.23 |
| LH_Thickness_rostralmiddlefrontal | 2.3 | 0.14 |
| LH_Thickness_superiorfrontal | 2.58 | 0.18 |
| LH_Thickness_superiorparietal | 2.12 | 0.14 |
| LH_Thickness_superiortemporal | 2.6 | 0.2 |
| LH_Thickness_supramarginal | 2.43 | 0.19 |
| LH_Thickness_temporalpole | 3.37 | 0.43 |
| LH_Thickness_transversetemporal | 2.24 | 0.26 |

Supplementary Table 3: The statistics for the image variables, which are the cortical thickness extracted from MR images.

| Image Variables | Mean | Standard Deviation |
|---|---|---|
| RH_Thickness_bankssts | 2.46 | 0.23 |
| RH_Thickness_caudalanteriorcingulate | 2.32 | 0.2 |
| RH_Thickness_caudalmiddlefrontal | 2.43 | 0.18 |
| RH_Thickness_cuneus | 1.8 | 0.15 |
| RH_Thickness_entorhinal | 3.05 | 0.38 |
| RH_Thickness_frontalpole | 2.64 | 0.25 |
| RH_Thickness_fusiform | 2.56 | 0.22 |
| RH_Thickness_inferiorparietal | 2.36 | 0.18 |
| RH_Thickness_inferiortemporal | 2.67 | 0.19 |
| RH_Thickness_insula | 2.84 | 0.24 |
| RH_Thickness_isthmuscingulate | 2.22 | 0.2 |
| RH_Thickness_lateraloccipital | 2.1 | 0.18 |
| RH_Thickness_lateralorbitofrontal | 2.55 | 0.19 |
| RH_Thickness_lingual | 1.89 | 0.17 |
| RH_Thickness_medialorbitofrontal | 2.36 | 0.17 |
| RH_Thickness_middletemporal | 2.73 | 0.2 |
| RH_Thickness_paracentral | 2.28 | 0.22 |
| RH_Thickness_parahippocampal | 2.48 | 0.24 |
| RH_Thickness_parsopercularis | 2.48 | 0.19 |
| RH_Thickness_parsorbitalis | 2.62 | 0.2 |
| RH_Thickness_parstriangularis | 2.35 | 0.17 |
| RH_Thickness_pericalcarine | 1.54 | 0.16 |
| RH_Thickness_postcentral | 2.02 | 0.15 |
| RH_Thickness_posteriorcingulate | 2.33 | 0.18 |
| RH_Thickness_precentral | 2.35 | 0.22 |
| RH_Thickness_precuneus | 2.27 | 0.19 |
| RH_Thickness_rostralanteriorcingulate | 2.67 | 0.23 |
| RH_Thickness_rostralmiddlefrontal | 2.29 | 0.14 |
| RH_Thickness_superiorfrontal | 2.56 | 0.18 |
| RH_Thickness_superiorparietal | 2.11 | 0.15 |
| RH_Thickness_superiortemporal | 2.64 | 0.21 |
| RH_Thickness_supramarginal | 2.44 | 0.17 |
| RH_Thickness_temporalpole | 3.47 | 0.42 |
| RH_Thickness_transversetemporal | 2.29 | 0.29 |

Supplementary Table 4: The statistics for the image variables, which are the cortical thickness extracted from MR images. Continued from Table 3.

## References


[1] Cox, D. R. Regression models and life-tables. *Journal of the Royal Statistical Society: Series B (Methodological) 34*, 2 (1972), 187–202.

[2] Haider, H., Hoehn, B., Davis, S., and Greiner, R. Effective ways to build and evaluate individual survival distributions. *J. Mach. Learn. Res. 21*, 85 (2020), 1–63.

[3] Ishwaran, H., Kogalur, U. B., Blackstone, E. H., and Lauer, M. S. Random survival forests. *The annals of applied statistics 2*, 3 (2008), 841–860.

[4] Wei, L.-J. The accelerated failure time model: a useful alternative to the cox regression model in survival analysis. *Statistics in medicine 11*, 14-15 (1992), 1871–1879.

[5] Yu, C.-N., Greiner, R., Lin, H.-C., and Baracos, V. Learning patient-specific cancer survival distributions as a sequence of dependent regressors. *Advances in neural information processing systems 24* (2011).
